# Computational Investigation of the Missense Mutations in *DHCR7* Gene Associated with Smith-Lemli-Opitz Syndrome

**DOI:** 10.3390/ijms19010141

**Published:** 2018-01-04

**Authors:** Yunhui Peng, Rebecca Myers, Wenxing Zhang, Emil Alexov

**Affiliations:** 1 Department of Physics and Astronomy, Clemson University, Clemson, SC 29630, USA; yunhuip@g.clemson.edu; 2 Department of Healthcare Genetics, Clemson University, Clemson, SC 29630, USA; rlmyers@g.clemson.edu; 3 Department of Chemistry, Clemson University, Clemson, SC 29630, USA; wenxinz@g.clemson.edu

**Keywords:** Smith-Lemli-Opitz syndrome, missense mutations, DHCR7, binding free energy, folding free energy, KNN classification, molecular dynamics simulation, MM/PBSA

## Abstract

Smith-Lemli-Opitz syndrome (SLOS) is a cholesterol synthesis disorder characterized by physical, mental, and behavioral symptoms. It is caused by mutations in 7-dehydroxycholesterolreductase gene (*DHCR7*) encoding DHCR7 protein, which is the rate-limiting enzyme in the cholesterol synthesis pathway. Here we demonstrate that pathogenic mutations in DHCR7 protein are located either within the transmembrane region or are near the ligand-binding site, and are highly conserved among species. In contrast, non-pathogenic mutations observed in the general population are located outside the transmembrane region and have different effects on the conformational dynamics of DHCR7. All together, these observations suggest that the non-classified mutation R228Q is pathogenic. Our analyses indicate that pathogenic effects may affect protein stability and dynamics and alter the binding affinity and flexibility of the binding site.

## 1. Introduction

Smith-Lemli-Opitz syndrome (SLOS) is an inherited disorder of cholesterol synthesis characterized by intellectual disability and multiple malformations, including facial and genital abnormalities and syndactyly and was first described by Smith and coworkers [1]. The reported incidence of SLOS varies widely depending on the heterogeneity of the population studied, the biochemical methods used and the alleles assessed. Current estimates of SLOS carrier frequency in Caucasian populations lie between 1% and 3% [2,3,4]. SLOS is more prevalent in individuals of northern and eastern European descent and is rarely described in individuals of Asian or African descent [5]. Reports that up to 80% of affected fetuses, likely those heterozygous for null mutations, die before birth and that milder cases of the disease may not be diagnosed, conceivably prevent accurate determination of frequency [6,7,8]. The majority of “classical” SLOS patients are compound heterozygotes with one severe null mutation and a second missense mutation which retains some enzyme functionality. Milder cases often possess two less severe missense mutations [9].

SLOS is linked to mutations in 7-dehydroxycholesterol reductase (DHCR7), which is the rate-limiting enzyme in the cholesterol synthesis pathway [10]. DHCR7 reduces the C7–C8 double bond of 7-dehydrocholesterol (7DHC), the precursor molecule to cholesterol [11]. Cholesterol, though harmful in high levels, is essential to life since it is involved in membrane structure and permeability, synthesis of steroid hormones and proper fetal development. The loss of functionality of the DHCR7 enzyme in individuals with SLOS results in a significant decrease in cholesterol levels and possibly toxic buildup of 7DHC and other cholesterol precursors [12]. It was shown that accumulation of 7DHC in the brains of rats is associated with intellectual and learning disabilities [13,14].

In addition to its role in cholesterol synthesis, 7DHC is also required for vitamin D3 production. Exposure to sunlight cleaves the C9–C10 bond of 7DHC in the skin, resulting in vitamin D3. Vitamin D3 is essential for calcium absorption and bone health [13]. As DHCR7 activity decreases the amount of 7DHC available for vitamin D3 synthesis, there is a potential heterozygote advantage to carriers of *DHCR7* mutations, which typically decrease enzymatic activity [14,15]. This may explain the prevalence of mutations originating in areas with decreased sun exposure such as northern Europe and northeast Asia [7,16].

The *DHCR7* gene maps to chromosome 11q13.2–13.5 [17,18,19] and consists of nine exons with the initiation codon located in exon three. The gene is expressed in all tissues with peak expression in adrenal glands, liver and brain [17]. *DHCR7* encodes a 475 amino acid polypeptide with a molecular weight of 54.5 kDa, which is a transmembrane protein located in the endoplasmic reticulum (ER) membrane, the location of cholesterol synthesis.

The first *DHCR7* mutations were identified in 1998 by several groups and the early years of the 21st century resulted in more advanced molecular tests to rapidly identify *DHCR7* mutations [17,18]. Most mutations are identified through sequence analysis of coding exons and flanking intronic sequences [5,17]. To date, more than 160 *DHCR7* mutations have been reported [5]. The most common mutation with a prevalence of ~30% of reported SLOS patients is the IVS8AS G > C − 1 splice acceptor site mutation. This results in the inclusion of 134 base pairs of intronic sequence into the transcript and a non-functional protein. Other common mutations include T93M, W151X, V326L and R404C. 

The majority of pathogenic *DHCR7* mutations occur in the highly conserved C-terminus region of the protein. In their molecular model of the DHCR7 protein, Li and coworkers predicted two overlapping binding sites: one for docking of the sterol 7DHC and one for binding of the coactivator NADPH [19]. As both binding sites are critical for proper protein function, it can be speculated that mutations affecting these areas would be most likely to result in disease. In support of this hypothesis, Waterham and Hennekam conducted a systematic review of published SLOS patients and compared genotype with phenotype [5]. They concluded that the most severely affected patients presented with two null alleles or two mutations in the 8–9 cytoplasmic loop while a milder phenotype was associated with mutations in the 1–2 loop or one mutation in the N- or C-terminus [5].

In the present study, we obtained variations in the *DHCR7* gene from online databases and modelled their effects on the corresponding protein to make predictions about SLOS phenotype. We demonstrate that structural and conservation properties are good discriminators between pathogenic and non-pathogenic mutations, while folding free energy changes (∆∆Gs) are not. This is consistent with previous observations [20] that current methodology for computing ∆∆Gs are not accurate enough when applied to membrane proteins. Furthermore, based on detailed analysis of selected mutants, we predict that the currently non-classified mutation, R228Q, is pathogenic.

## 2. Results and Discussion

### 2.1. Mapping Missense Mutations onto the 3D Structure of DHCR7 Protein

**Figure 1:**
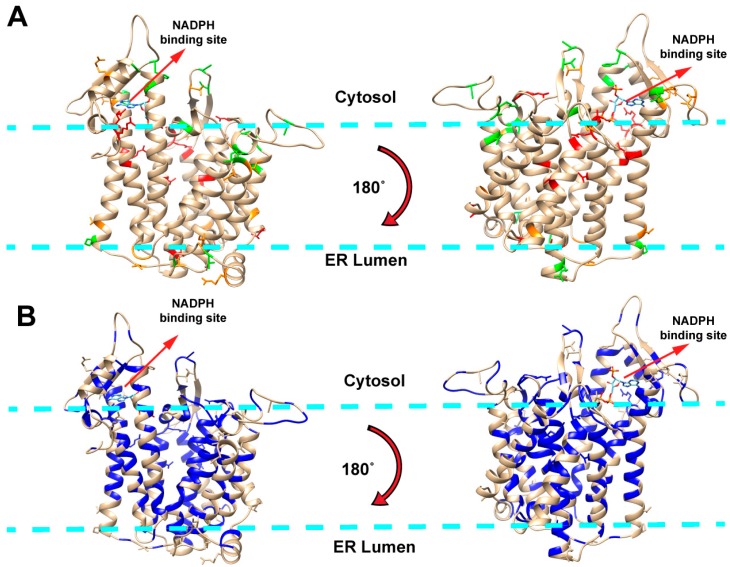
(**A**) Visualization of mutations mapped onto DHCR7 protein. Red, orange and green colored sites represent pathogenic, unknown effects and non-pathogenic mutations, respectively. The membrane boundaries are schematically shown with light blue dashed lines; (**B**) most highly evolutionarily conserved residues mapped onto DHCR7 protein. Residues with EC score > 0.9 are marked with blue and all mutation-affected residues are shown with side chain. The membrane boundaries are schematically shown with light blue dashed lines.

The dataset of *DHCR7* missense mutations includes three types of mutations: pathogenic, non-pathogenic and mutations of unknown effect. The mutations were visualized by mapping them onto the DHCR7 structure (Figure 1A). Pathogenic mutations are predominantly located in transmembrane and ligand-binding regions while non-pathogenic mutations are primarily situated outside the membrane. This observation indicates that pathogenic mutations occur at protein sites that are either buried or directly involved in protein function, which corroborates the findings of previous investigations [21,22,23,24]. To investigate the linkage between structural and evolutionary features of DHCR7 protein, we obtained the evolutionary conservation score (EC score) for each residue from multiple sequence alignment and mapped them onto the 3D structure of DHCR7 (Figure 1B). The transmembrane and ligand-binding regions appear to be highly conserved. Thus, most pathogenic mutations are located in highly conserved positions, while non-pathogenic mutations are less conserved. To further quantitatively assess the mutations’ effects, we computed the relative solvent accessible surface area (rSASA), evolutionary conservation score (EC score) and folding free energy change (∆∆G) for all mutations studied in this work (Table S1). Pathogenic mutations tend to have lower rSASA values and higher EC scores compared with non-pathogenic mutations. However, ∆∆G results show no obvious tendency to discriminate pathogenic from non-pathogenic mutations. The predictions made with different servers frequently contradict each other resulting in large standard deviation (SD) when averaging these predictions (Table S1). As DHCR7 is a transmembrane protein and recent work [20] demonstrated that current tools of ∆∆G predictions are not accurate when applied to membrane proteins, this may explain why ∆∆G fails to discriminate pathogenic from non-pathogenic mutations in this case. In addition, we also performed Polyphen predictions on all types of mutations (Table S1). Almost all the pathogenic mutations are predicted to be probably damaging by Polyphen. However, Polyphen overestimated the deleteriousness of the non-pathogenic mutations. About half of the non-pathogenic mutations were classified as possibly or probably damaging. Thus, Polyphen has limited accuracy in discriminating the pathogenic mutations from the mutations with unknown effects for this particular protein.

### 2.2. Classification of the Mutations with Unknown Effects Using KNN Model

One of the goals of this study was to identify biophysical features allowing us to distinguish between pathogenic and non-pathogenic mutations, and thus to make predictions about unclassified mutations. Above, we outlined several biophysical features, namely rSASA, EC score, PD and ΔΔG, which will be used in conjunction with the K-nearest neighbors (KNN) method (see Method section). The dataset includes 16 pathogenic mutations and 23 non-pathogenic mutations. These 39 mutations were randomly partitioned into training dataset (29 mutations) and test dataset (10 mutations) and then subjected to the KNN classifications. As the ΔΔG was shown to be less successful in distinguishing between pathogenic and non-pathogenic mutations, we performed the KNN classification with and without the ΔΔG (Table S2). The classification shows better performance without using the ΔΔG and the accuracy is 100% when K value is within 5 to 9. Here, we select *K* = 7 (the median of the *K* value corresponding to highest accuracy). Finally, KNN model with *K* = 7 and using properties: rSASA, EC score and PD applied to classify the mutations with unknown effects (Table 1). Thus, we predict that among all currently known unclassified mutations, only R228Q is pathogenic. In Table 1 we also compared our KNN classification results with the predictions from Polyphen. Consistent with our results, Polyphen predicted R228Q to be probably damaging. However, Polyphen gives contradictory predictions for eight additional mutations (predicted to be probably damaging), which are classified as non-pathogenic by our KNN classification. Overestimation of mutation deleteriousness was also observed when applying Polyphen to the known non-pathogenic mutations (Table S1).

### 2.3 Case Study of Selected Mutations Using Molecular Dynamics (MD) Simulations

The above classification and analyses were performed using fast computational approaches and were applied to the entire dataset. We selected a subset of mutations for extensive MD simulations to investigate the possibility that pathogenic and non-pathogenic mutations have different effects on DHCR7 protein conformational dynamics. For this purpose, we selected 10 representative mutations including five pathogenic mutations (T154R, E288K, T289I, G303R and R404C), two non-pathogenic mutations (R260Q and A452T) and three mutations with unknown effects (V134L, R228Q and F361L). These mutations are localized to different regions of protein structure. Five mutations (T154R, R228Q, E288K, T289I and G303R) are located in the transmembrane region and are buried in the membrane, two mutations (F361 and R404C) occur near the ligand-binding site and potentially affect ligand binding, and the remaining three mutations (V134, R260Q and A452T) are in neither the transmembrane region nor the ligand binding site.

**Figure 2:**
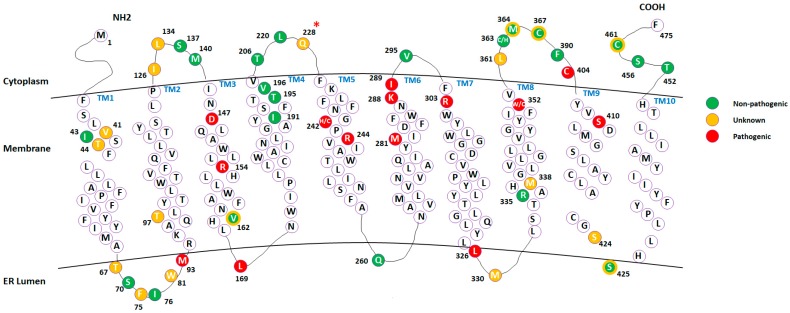
The topology of the cytosol loops (CL), the C terminal domain (CTD) and transmembrane domains (TM) in DHCR7 structure. Mutation sites are mapped with different colors according to mutation type (double color is applied for sites with unknown and non-pathologic classification). The unclassified mutation R228Q, which we predict to be pathogenic, is highlighted with a red asterisk.

Since our focus was on protein conformational dynamics, we calculated the corresponding RMSDs and RMSFs for the wild type and mutant proteins. The average RMSD data shows no obvious difference between wild type protein and proteins with non-pathogenic or pathogenic mutations. However, the average RMSF indicates some differences between the wild type and mutants. For example, in the mutant A452T, cytosol loops (CL) 2 and 4 and transmembrane domain (TM) 10 regions are more rigid compared to the wild type (Table 2). However, no apparent patterns were identified to differentiate pathogenic mutations and non-pathogenic mutations by simply observing the graphs. A previous study of the AGAL protein has indicated a correlation between the protein’s flexibility and the severity of a mutant’s pathogenicity [25]. Thus, to identify such potential correlation in DHCR7 protein, we mapped the pathogenic and non-pathogenic mutations on the average RMSF of the wild type proteins (shown in Figure S1). We observed that most pathogenic mutations are located on the low RMSF region while the non-pathogenic mutations show the opposite trend. As the low RMSF residues are mostly transmembrane, such observed correlation is expected when majority of the pathogenic mutations are located on the transmembrane region. In addition, further analysis was performed by grouping the residues into different regions and then summing up the RMSF of residues in that region to get a region-RMSF. Based on DHCR7 protein structure information [26], residues were grouped into regions: TM1 (residues 40–60), TM2 (residues 94–115), TM3 (residues 145–164), TM4 (residues 176–191), TM5 (residues 235–256), TM6 (residues 268–288), TM7 (residues 302–326), TM8 (residues 332–352), TM9-10 (residues 408–442), CL1 (residues 116–144), CL2 (residues 198–234), CL3 (residues 289–301), CL4 (residues 354–407) and CTD (residues 443–475). The topology of the cytosol loops (CL), the C terminal domain (CTD) and transmembrane domains (TM) mapped with selected mutations are further represented for better visualization of the DHCR7 structure (Figure 2).

Table 2 shows the region-RMSFs. Pathogenic mutations tend to decrease the flexibility in the TM1, TM2 and CL2 regions and increase the flexibility in the TM7 and TM9_10 regions. Very little is known about DHCR7 function and structural changes occurring during chemical reactions, so we used the above observation to suggest an empirical formula that discriminates between pathogenic and non-pathogenic mutations, which were subjected to MD simulations (ideally, one should perform such an analysis for mutations analyzed in this manuscript, but this is too computationally demanding). For the wild type and each mutant, we sum the RMSFs of TM1, TM2 and CL2 and then subtract the RMSFs of TM7 and TM9_10 (last column in Table 2). We refer to this quantity as cumulative RMSF. The wild type and non-pathogenic mutants have cumulative RMSFs larger than 50 Å while all pathogenic mutants have a cumulative RMSF less than or equal to 46 Å. Among non-classified mutations, V134L is confirmed to be non-pathogenic, while R228Q and F361L show the same cumulative RMSFs as pathogenic mutations. Thus, it is encouraging to observe that R228Q is independently confirmed to be pathogenic mutation (see KNN classification above), while F361L cannot be classified with high confidence and additional investigations are reported in the next section. 

### 2.4. Analysis of Mutations’ Pathogenic Effects

#### 2.4.1. Ligand Binding

**Figure 3:**
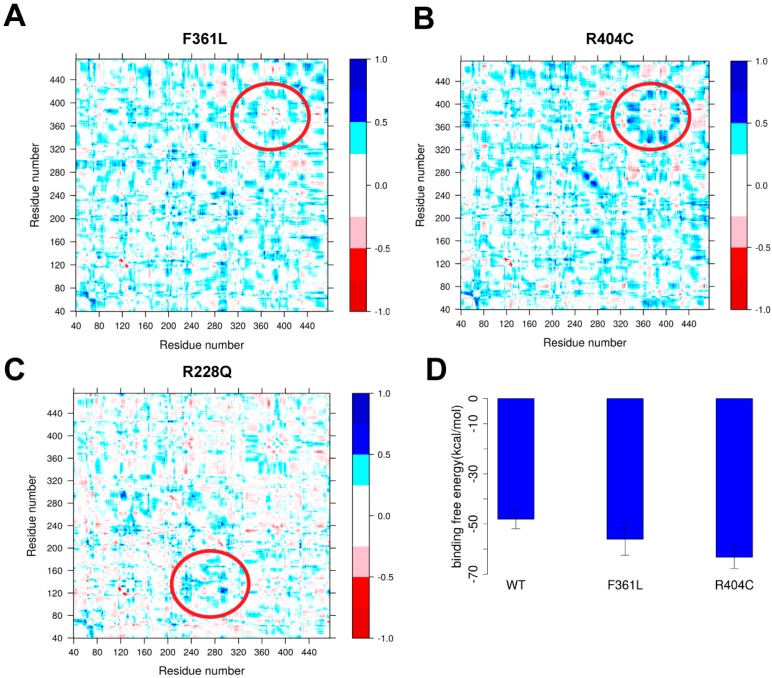
(**A**–**C**) The changes in residue cross-correlation for mutations F361L, R404C and R228Q; (**D**) NADHP binding free energy for WT and mutations F361L and R404C.

Here, we investigated the possibility that mutations may change DHCR7 functionality by altering the binding affinity towards its ligand NADPH. For this purpose, we compared the effects of F361L (non-classified) and R404C (pathogenic mutation), both located near the NADPH binding site. It is anticipated that NADPH binding will cause structural rearrangement of the binding site and the conformational flexibility of the binding pocket is essential for proper protein function. We tested the effects of F361L and R404C on binding pocket flexibility by comparing them with the wild type protein. This was done using the MD trajectories obtained above and computing the residue cross-correlation for each trajectory with Bio3D [27]. These types of analyses were successfully used to elucidate the effects of a single mutation on the human β2-microglobulin’s protein dynamics [28]. For each mutation and wild type, we calculated the average cross-correlation from three independent MD runs. Finally, the residue cross-correlation changes for mutations F361L and R404C are shown in Figure 3A,B, which is the subtraction of the averaged cross-correlation map between mutant and wild type proteins. Significant changes of the cross-correlation coefficient near the NADPH binding site were found for R404C, highlighted with a circle in Figure 2, but not for F361L.

We also performed MM/PBSA analysis to investigate the effect of mutations on NADPH binding affinity (Figure 3D). Mutation R404C results in a large increase of the binding affinity by about 15 kcal/mol. As shown in the literature [21,29,30], any large deviation from wild type characteristics may be deleterious. In this case, R404C mutations contribute to disease by altering the binding affinity of NADPH. Compared to the effect of F361L, we observe that binding affinity is much less affected. This, combined with correlation analysis, allows us to speculate that F361L is a non-pathogenic mutation.

#### 2.4.2. Protein Dynamics

We further analyzed the selected mutations including our predicted pathogenic mutation R228Q to identify other pathogenic effects on protein functionality. The residue cross-correlation analysis of R228Q (Figure 2C) indicates a local conformational change near the mutation site. The R228Q mutation makes the corresponding region more rigid, resulting in local flexibility changes in CL2. Changes in protein dynamics are also observed in the residue cross-correlation analysis of other pathogenic mutations such as E288K and G303R (shown in Figure S2), indicating that alterations in DHCR7 protein dynamics likely contribute to protein dysfunction. 

### 2.5. Allele Frequency Analysis

**Figure 4:**
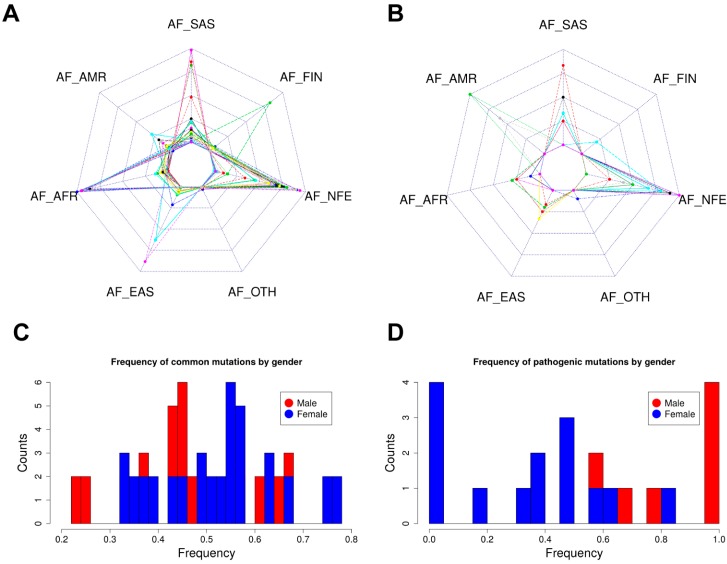
The frequency distribution of *DHCR7* mutations. AFR, AMR, EAS, FIN, NFE, SAS and OTH represent African and African American, American, East Asian, Finnish, Non-Finnish European, South Asian and other populations, respectively. (**A**) The frequency distribution among different populations of the top 40 DHCR7 mutations of varying types occurring in more than 50 individuals archived in the ExAC database; (**B**) The frequency distribution among different populations of pathogenic missense mutations chosen for this study; (**C**) The frequency distribution in males and females of the top 40 DHCR7 mutations of varying types occurring in more than 50 individuals archived in the ExAC database; (**D**) The frequency distribution in males and females of pathogenic missense mutations chosen for this study.

We compared the frequency distribution of pathogenic mutations and frequently-occurring common mutations among different populations and genders. Figure 4A displays the top 40 *DHCR7* mutations of varying types occurring in more than 50 individuals archived in the ExAC database. At the same time, Figure 3B shows the distribution of pathogenic missense mutations chosen for this study within the same set of populations. The most frequently-occurring mutations in the general population are found in individuals of non-Finnish European descent followed by South Asian and African and African American descent (Figure 4A). Additionally, individuals of non-Finnish European and South Asian descent have the highest frequency of pathogenic mutations as shown in Figure 4B. African and African American populations have few cases of SLOS despite high occurrences of *DHCR7* mutations. The low occurrence and frequency of mutations in Europeans of Finnish descent is supported by the extremely low number of SLOS cases in Finland [31].

Interestingly, females in the overall ExAC population possess more *DHCR7* mutations at higher frequencies than males (Figure 4C), while this is an opposite for the pathogenic mutations investigated in this manuscript (Figure 4D), though no support for this trend has been found in the literature. One can speculate that this is linked to sex hormones and is embryo lethal, but the observation that females carry more pathogenic mutations than males should be taken with precaution.

## 3. Materials and Methods

### 3.1. Selection of DHCR7 Missense Variants

The missense mutations investigated in this work were selected using ClinVar [32] and ExAC [33] databases. The ClinVar database (https://www.ncbi.nlm.nih.gov/clinvar/) was queried using the search term “DHCR7”. The results were further refined by missense mutations consisting of benign (2), likely benign (3), uncertain significance (30), likely pathogenic (15), pathogenic (26) and conflicting reports of pathogenicity (3) (as of 13 November 2017). The ExAC (Exome Aggregation Consortium) Browser (http://exac.broadinstitute.org/) was queried using the search term “DHCR7” and the entries were sorted by allele frequencies in descending order. The missense variants with an allele frequency greater than 0.00001, which were also classified in ClinVar were chosen for further in silico analysis. Of the chosen mutations, the variants defined as pathogenic or likely pathogenic in Clinvar database are classified as pathogenic mutations in this study while the others defined as uncertain significance in Clinvar database are classified as mutations with unknown effects. E288K and G303R are previously reported SLOS-causing mutations [34,35] although they are not classified as pathogenic in the Clinvar database. Thus, E288K and G303R were treated as pathogenic mutations in this study. Overall, 16 pathogenic mutations and 18 mutations with unknown effects are classified for this study.

### 3.2. Selection of Non-Pathogenic DHCR7 Mutations

We first obtained the missense mutations in *DHCR7* gene from the ExAC database [33], including the whole genome sequencing data from 60,706 unrelated individuals. In total, 280 missense mutations in *DHCR7* were identified. The ExAC database also provides the corresponding allele frequency data from the 1000 Genomes Project and the NHLBI-GO Exome Sequencing Project (ESP) for each mutation. Individuals participating in the 1000 Genomes Project were all healthy while the objective of the ESP is discovery of novel genes and mechanisms contributing to heart, lung and blood disorders. As our goal was to select non-pathogenic mutations from the ExAC database, we applied the following selection criteria: (a) mutations with allele frequency > 0 in the 1000 Genomes Project; (b) mutations with allele frequency of 0 in the ESP. Thus, we classified the mutations identified from the healthy population of 1000 Genomes Project but not from the ESP as non-pathogenic mutations in this study. In total, 23 non-pathogenic missense mutations were identified.

### 3.3. Obtaining Allele Frequency and Gender Occurrence

The allele frequency and gender data of *DHCR7* mutations were obtained from EXAC database [33]. The most recent database version was downloaded from the FTP site (http://exac.broadinstitute.org/downloads) and mutations affecting the DHCR7 protein as well as their corresponding allele frequencies and gender data were obtained. The frequency of mutation by gender is calculated by the number of carrier females or males divided by the total number of carrier individuals.

### 3.4. Generation of the 3D Model for DHCR7

The 3D structure of the DHCR7 protein was generated by homology modeling due to lack of an existing experimental structure. Structure of the integral membrane sterol reductase from *Methylomicrobium alcaliphilum* (PDB: 4QUV) [19] was used as a template and subjected to MODELLER [36] for homology modeling. The sequence identity between the template and DHCR7 is 37% (sequence alignment is shown in Figure S3) and thus high structural similarity was observed between the generated model and template. The model with lowest DOPE score was selected for this study and further subjected to automatic loop refinement with MODELLER [36].

### 3.5. Property Distance (PD)

**Figure 5:**
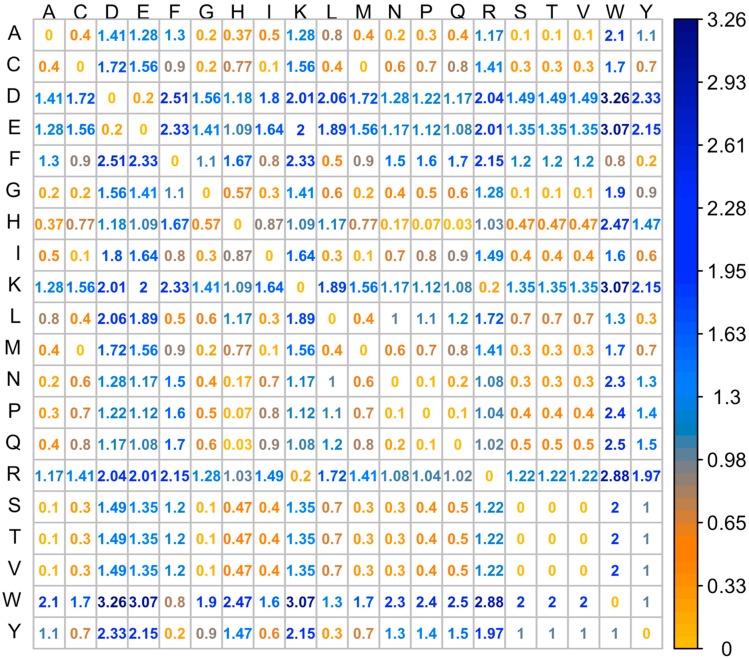
Property distance for all types of amino acid pairs.

To quantify the physical-chemical property differences between the wild type and mutant residues, we used the property distance (PD) as a parameter to quantitatively describe such changes. In this study, we describe physical-chemical properties of a particular residue using a property vector which includes two elements: hydrophobicity and charge. The hydrophobicity of the residues are taken from an experimentally determined hydrophobicity scale [37,38]. R and K carry +1 charges while E and D have −1 charges. All other residues are considered neutral. PD represents the Euclidean distance of the property vector between the wild type and mutant residues (shown in Equation (1)). The *PD* between all types of residues are shown as a matrix in Figure 5.

(1) $$PD\left(x,y\right)=\sqrt{{\left(H\left(x\right)-H\left(y\right)\right)}^{2}-{\left(Q\left(x\right)-Q\left(y\right)\right)}^{2}}$$

where *x* and *y* represent two types of residues; *H* and *Q* are corresponding hydrophobicity and charge for a particular residue.

### 3.6. Evolutionary Conservation Score (EC Score) Calculation

The DHCR7 sequence from 35 different species were collected from UnitProt [39] and subjected to multiple sequence alignment with the T-Coffee webserver [40]. The EC score of each residue in the human DHCR7 sequence was calculated using the multiple sequence alignment with the following equation:

(2) $$EC\;score\left(i\right)=\frac{N{\left(i\right)}_{identity}}{N{\left(i\right)}_{total}}$$

where $N{\left(i\right)}_{identity}$ is the number of the species sharing identical residues in position *i* of the human DHCR7 sequence and $N{\left(i\right)}_{total}$ is the total number of the species in the multiple sequence alignment.

### 3.7. Folding Free Energy Change (ΔΔG) and Relative Solvent Accessible Surface Area (rSASA) Calculation

Several webservers were used to predict the effect of mutations on protein stability (folding free energy change (∆∆G)) using the generated homology model of DHCR7 protein. The webservers used in this study include DUET [41], Eris [42], mCSM [43], SDM [44], Foldx [45] and SAAFEC [46]. The SASA were calculated using VMD [47]. As DHCR7 is a transmembrane protein, the membrane was also included when calculating the SASA. Thus, only the amino acids exposed to water were treated as exposed and the transmembrane regions were treated as buried in the calculation. The rSASA for residues were calculated using the following equation:

(3) $$rSASA\left(i\right)=\frac{SASA\left(i\right)}{SASA{\left(i\right)}_{max}}$$

where *SASA*(*i*) is the SASA measured for particular residue *i* and $SASA{\left(i\right)}_{max}$ is the maximum SASA obtained for a free residue (entire residue taken off the protein). 

### 3.8. Molecular Dynamic Simulations

The membrane-protein-ligand system was built primarily using the CHARMM-GUI [48] tools. The DHCR7 protein with ligand structure was obtained from previous homology modeling. Ten mutant (V134L, T154R, R228Q, R260Q, E288K, T289I, F361L, G303R, R404C and A452T) structures were derived from the wild type DHCR7 protein structure using VMD 1.9.3 [47] mutator package. The protein was embedded in a POPC bilayer using the CHARMM-GUI website. The protein was oriented to align with 4QUV structure in the OPM [49] database. When the oriented protein was placed into the membrane, the z axis of the protein matched the z axis of the membrane. The whole system was solvated with 0.15 M KCl. The final system was 89.13 × 89.13 × 96.64 Å^3^ with a total of about 70,800 atoms.

Molecular dynamic simulation (MDS) was performed using NAMD2.11 [50]. The system first underwent energy minimization for 10 ps, then equilibrated through 6 cycles where harmonic constraints were applied to keep original positions of: (a) lipid head groups (force constants were gradually reduced from 5 kcal∙mol^−1^∙Å^−2^ to 0 kcal∙mol^−1^∙Å^−2^), (b) protein backbone (force constants were gradually reduced from 10 kcal∙mol^−1^∙Å^−2^ to 0 kcal∙mol^−1^∙Å^−2^), and (c) protein sidechains (force constants were gradually reduced from 5 kcal∙mol^−1^∙Å^−2^ to 0 kcal∙mol^−1^∙Å^−2^). In addition, dihedral restraints were applied to keep cis double bonds and c2 chirality (force constants were gradually reduced from 500 kcal∙mol^−1^∙Å^−2^ to 0 kcal∙mol^−1^∙Å^−2^). A 1 fs timestep was used in the first few cycles and then switched to 2 fs for wild type whereas much smaller timesteps such as 0.01 fs were used for mutants to prevent restraints from failing. In the first two cycles, NVT simulation was performed and then switched to NPT simulation in the later cycles. Temperature was held at 303.15 K using a Langevin thermostat with a damping coefficient of 10 ps^−1^ and velocity rescaling thermostat. The pressure was maintained at 1 atm using a Langevin piston barostat with an oscillation period of 50 fs and a damping time constant of 25 fs. Electrostatic interactions between charged atoms were calculated using the particle mesh Ewald method. Van der Waals interactions were truncated at 12 Å with a switching function applied from 10 Å. RATTLE is used to constrain the length of all bonds involving a hydrogen atom. This stage of equilibration lasts for tens of ps to hundreds of ps. Then three 10 ns equilibration and 10 ns production runs with no constraints were performed for the wild type and each mutant. A 2 fs timestep was used. No velocity rescaling thermostat was used. Other conditions are the same as the previous stage. RMSD and root mean square fluctuation (RMSF) with the structure at the beginning of the 10 ns run as the reference structure were calculated using VMD 1.9.3.

### 3.9. MM/PBSA Analysis

To estimate the binding affinity of the DHCR7 protein with the ligand NADPH, we calculated the binding free energy using the MM/PBSA approach. For this purpose, we performed three independent 20 ns MD simulations as described above. We took the frames with an interval of 20 ps from the last 10 ns and a total of 500 frames were selected from each trajectory. All ions, water and lipids were removed before MM/PBSA energy calculations. All the energy terms were averaged over 500 frames for each trajectory and the mean and standard deviation of binding free energy were calculated for wild type and mutant structures. The internal energy and van der Waals interactions were calculated using NAMD2.11b [50] by subjecting the structure to a one step equilibration at 300 K using dielectric constant = 2 for protein and = 80 for solvent. The electrostatic components of the binding free energy (Coulombic and solvation energy) were calculated by solving the Poisson Boltzmann (PB) equation using the Delphi program [51] with dielectric constant = 2 for protein and = 80 for solvent. The solvent accessible surface area (SASA) was calculated by VMD [47] with the solvent and lipid. The non-polar component of the solvation was further calculated with the following widely-used equation:

(4) $$G_{SASA}=\alpha \cdot SASA+\beta$$

where *α* = 0.0054 and *β* = 0.92 kcal/mol.

### 3.10. K-Nearest Neighbors (KNN) Classification

K-Nearest Neighbors algorithm was used to classify the missense mutations with unknown effects in DHCR7 protein. The dataset includes 16 pathogenic missense mutations and 23 non-pathogenic missense mutations (non-classified/unknown effect mutations were excluded). The dataset was randomly partitioned into a training dataset (29 mutations) and a testing dataset (10 mutations). The KNN classification was performed using R program and various numbers of K values were tested to obtain the best performance.

## 4. Conclusions

We investigated the effects of mutations causing SLOS on the biophysical characteristics of DHCR7 protein with the goal of identifying methods allowing the discrimination of pathogenic mutations from non-pathogenic mutations. We found that pathogenic mutations are located either within the transmembrane region or are near the ligand-binding site and are highly conserved between species. In contrast, non-pathogenic mutations observed in the general population are located outside the transmembrane region and have different effects on the conformational dynamics of DHCR7. Our analyses confirmed the inability of folding free energy modeling to deliver reliable results and to be used to discriminate pathogenic from non-pathogenic mutations in membrane proteins. Future investigations may include modeling the effects of *DHCR7* mutations on melting temperature (Tm) via MD simulations conducted at different temperatures using the methodology adopted from recent work on NBD1 domain [52]. As mentioned in the work of Estacio et al. [52], the decrease of Tm may cause the protein to adopt partially misfolded states that become targeted for degradation.

In this work, using three characteristics: solvent exposure of the mutation site, residue conservation and physico-chemical descriptors, we were able to distinguish between pathogenic and non-pathogenic mutations. This observation, along with extensive MD simulations and MM/PBSA modeling, was used to classify R228Q as a pathogenic mutation.

Taken together, these observations suggest that the non-classified mutation R228Q is in fact pathogenic. The analyses performed indicate that pathogenic effects may be of different origin, from affecting protein stability and dynamics to altering binding affinity and flexibility of the binding site. 

## Figures and Tables

**Table 1:** KNN classifications and Polyphen predictions of the mutations with unknown effects. P and N represent pathogenic and non-pathogenic mutations, respectively.

Mutation	KNN Classification	Polyphen	Mutation	KNN Classification	Polyphen
A41V	N	Benign	R228Q	P	Probably damaging
I44T	N	Benign	V330M	N	Probably damaging
A67T	N	Possibly damaging	V338M	N	Benign
I75F	N	Benign	F361L	N	Probably damaging
R81W	N	Probably damaging	T364M	N	Probably damaging
A97T	N	Possibly damaging	R367C	N	Probably damaging
V126I	N	Probably damaging	G424S	N	Probably damaging
V134L	N	Benign	G425S	N	Benign
A162V	N	Possibly damaging	R461C	N	Probably damaging

**Table 2:** RMSF values per structural region (see text for details) for each of the mutants. The RMSFs are given in Å units. The last column reports the RMSF calculated as the sum of RMSFs of TM1, TM2 and CL2 subtracted by RMSF of TM7 and TM9_10. Values larger than 50 Å are underlined.

**Pathogenic Missense Mutations**															
	TM1	TM2	TM3	TM4	TM5	TM6	TM7	TM8	TM9_10	CL1	CL2	CL3	CL4	CTD	TM1+TM2-TM7-TM9_10+CL2
T154R	22.9	17.6	15.5	9.1	14.5	17.4	17.2	17.8	31.6	53.1	48.6	8.8	78.8	30.8	40.3
E288K	19.6	16.4	16.0	10.5	13.1	14.3	16.0	18.4	26.1	49.2	38.6	13.6	77.5	32.0	32.7
T289I	25.2	18.0	17.8	12.8	14.1	14.3	19.5	17.5	28.7	50.1	47.9	10.1	71.1	30.4	42.9
G303R	21.1	18.9	16.8	11.0	13.3	16.0	18.2	17.0	30.2	50.5	49.2	10.4	65.0	30.1	40.9
R404C	23.4	16.5	16.0	10.8	16.0	16.8	20.8	23.3	31.6	48.9	57.4	10.0	80.1	32.7	44.9
**Missense Mutations with Unknown Effects**															
	TM1	TM2	TM3	TM4	TM5	TM6	TM7	TM8	TM9_10	CL1	CL2	CL3	CL4	CTD	TM1+TM2-TM7-TM9_10+CL2
V134L	20.1	21.1	18.8	11.0	14.7	13.6	16.6	16.2	27.7	52.9	53.4	11.0	79.7	35.6	50.3
R228Q	17.6	17.0	15.9	8.5	13.6	13.0	15.6	16.9	27.6	53.2	54.2	10.7	75.8	36.7	45.6
F361L	19.4	17.4	14.8	9.9	14.2	14.0	18.3	16.6	28.8	54.7	50.8	11.7	74.8	33.5	40.5
**Non-Pathogenic Missense Mutations**															
	TM1	TM2	TM3	TM4	TM5	TM6	TM7	TM8	TM9_10	CL1	CL2	CL3	CL4	CTD	TM1+TM2-TM7-TM9_10+CL2
R260Q	19.7	18.6	15.5	9.4	12.9	14.6	15.4	17.2	24.4	58.4	52.1	11.4	79.3	28.1	50.6
A452T	20.9	19.6	17.8	10.5	13.6	16.2	16.4	17.2	26.0	55.2	52.8	8.7	66.6	30.1	51.0
**Wild Type**															
	TM1	TM2	TM3	TM4	TM5	TM6	TM7	TM8	TM9_10	CL1	CL2	CL3	CL4	CTD	TM1+TM2-TM7-TM9_10+CL2
WT	18.2	18.3	17.9	10.7	16.3	16.0	18.5	18.1	31.1	51.9	65.1	13.0	80.4	37.8	52.0

## Supplementary Materials

Supplementary materials can be found at www.mdpi.com/1422-0067/19/1/141/s1.

## Abbreviations

SLOS	Smith-Lemli-Opitz syndrome
DHCR7	7-dehydroxycholesterol reductase
ER	Endoplasmic reticulum
ExAC	Exome Aggregation Consortium
PD	Property distance
ECS	Evolutional conservation score
RMSD	Root mean square deviation
RMSF	Root mean square fluctuation
SASA	Solvent accessible surface area
rSASA	Relative solvent accessible surface area
KNN	K-Nearest Neighbors
MD	Molecules dynamics
CL	Cytosol loops
TM	Transmembrane domain
ESP	Exome Sequencing Project
