# Supplementary material for: Computational Investigation of the Missense Mutations in DHCR7 Gene Associated with Smith-Lemli-Opitz Syndrome
Source: Int J Mol Sci. 2018 Jan 4;19(1):141. doi: 10.3390/ijms19010141 (PMC5796090; doi:10.3390/ijms19010141)
Supplement: Supplementary file 1 [file ijms-19-00141-s001.pdf]

Table S1. Folding free energy, rSASA and Polyphen predictions for the mutations in DHCR7 protein. ΔΔGs are shown in kcal/mol and average ΔΔG are also calculated using the results from multiple webservers. Mutations A206T and H390T are located on the loop, not present in the template, thus the corresponding rSASA is highlighted in red to indicate low confidence for these two mutations.

**Pathogenic missense mutations**									
**Mutation**	**rSASA_mem**	**CV score**	**SAFFEC**	**mCSM**	**SDM**	**DUET**	**FOLDX**	**ΔΔG_ave**	**Polyphen**
**T93M**	0.21	0.62	0.53	0.10	0.30	-0.03	-0.52	0.08	Probably damaging
**G147D**	0.01	1.00	3.30	-1.53	-0.35	-1.39	7.40	1.49	Probably damaging
**T154R**	0.01	0.97	-3.55	-0.59	-1.45	-0.60	1.62	-0.91	Probably damaging
**S169L**	0.31	0.85	1.39	-0.25	0.69	-0.25	0.62	0.44	Probably damaging
**R242H**	0.02	1.00	0.17	-2.38	-0.05	-2.59	-0.20	-1.01	Probably damaging
**R242C**	0.02	1.00	0.40	-1.96	0.39	-2.05	-3.95	-1.43	Probably damaging
**G244R**	0.01	1.00	0.96	-1.39	-0.10	-1.20	4.72	0.60	Probably damaging
**V281M**	0.02	0.91	-0.19	-0.28	-0.08	-0.30	0.40	-0.09	Probably damaging
**E288K**	0.10	1.00	-15.77	-0.27	-1.17	-0.24	0.78	-3.33	Probably damaging
**T289I**	0.43	0.29	-0.60	-0.09	1.26	0.18	-0.70	0.01	Possibly damaging
**G303R**	0.04	1.00	-1.48	-1.15	-2.73	-1.22	21.73	3.03	Probably damaging
**V326L**	0.00	0.94	0.24	-1.08	0.20	-1.00	1.01	-0.13	Benign
**R352W**	0.06	0.94	0.00	-0.37	2.57	-0.55	0.12	0.35	Probably damaging
**R352Q**	0.06	0.94	-0.91	-0.81	-0.63	-0.81	0.60	-0.51	Probably damaging
**R404C**	0.04	0.97	-0.05	-1.97	0.11	-2.18	3.00	-0.22	Probably damaging
**G410S**	0.01	0.91	-2.71	-1.81	-0.55	-1.69	11.05	0.86	Probably damaging
**Missense mutations with unknown effects**									
**Mutation**	**rSASA_mem**	**CV score**	**SAFFEC**	**mCSM**	**SDM**	**DUET**	**FOLDX**	**Average**	**SD**
**A41V**	0.05	0.44	0.03	-0.28	-0.29	0.01	-0.30	-0.17	Benign
**I44T**	0.18	0.76	0.69	-1.29	-2.79	-1.19	1.40	-0.64	Benign
**A67T**	0.00	0.18	2.48	-1.59	-0.79	-1.66	5.65	0.82	Possibly damaging
**I75F**	0.53	0.24	-0.47	-0.64	0.12	-0.61	-0.56	-0.43	Benign
**R81W**	0.51	0.21	-0.55	-0.49	1.18	-0.58	-0.64	-0.22	Probably damaging
**A97T**	0.24	0.80	0.18	-1.28	-2.29	-1.19	1.64	-0.59	Possibly damaging
**V126I**	0.12	0.76	0.60	-0.78	0.74	-0.72	0.04	-0.02	Probably damaging
**V134L**	0.28	0.32	1.36	-0.63	1.13	-0.43	-1.24	0.04	Benign
**A162V**	0.13	0.76	1.18	0.00	0.68	0.19	-1.50	0.11	Possibly damaging
**R228Q**	0.18	0.97	1.27	-1.01	-0.84	-1.13	-0.23	-0.39	Probably damaging
**V330M**	0.58	0.85	-2.47	-0.52	-0.51	-0.45	-0.57	-0.90	Probably damaging
**V338M**	0.25	0.41	-0.92	-0.38	0.80	-0.19	-0.70	-0.28	Benign
**F361L**	0.01	0.91	-0.16	-2.72	-0.17	-2.80	-0.90	-1.35	Probably damaging
**T364M**	0.25	0.85	-0.63	-0.03	0.30	-0.32	-0.60	-0.26	Probably damaging
**R367C**	0.56	0.32	4.01	-0.37	0.83	-0.30	0.30	0.89	Probably damaging
**G424S**	0.08	0.35	-0.48	-0.48	2.60	-0.02	-0.03	0.32	Probably damaging
**G425S**	0.12	0.56	-0.95	-0.68	2.54	-0.28	-0.10	0.11	Probably damaging
**R461C**	0.54	0.71	3.02	-0.39	0.16	-0.31	1.25	0.75	Probably damaging
**Non-pathogenic missense mutations**									
**Mutation**	**rSASA_mem**	**Conser**	**SAFFEC**	**mCSM**	**SDM**	**DUET**	**FOLDX**	**Average**	**SD**
**V43I**	0.16	0.74	0.02	-0.32	0.59	-0.10	0.61	0.16	Benign
**G70S**	0.00	0.12	0.67	-1.68	-2.79	-1.95	2.51	-0.65	Benign
**V76I**	0.43	0.06	-1.00	-0.27	0.01	-0.19	0.75	-0.14	Benign
**A137S**	0.68	0.97	0.39	-0.81	-1.42	-0.66	-0.19	-0.54	Possibly damaging
**V140M**	0.37	0.53	-0.75	-0.49	-0.74	-0.59	-0.81	-0.67	Benign
**A162V**	0.13	0.76	0.94	0.00	0.68	0.19	-1.47	0.07	Possibly damaging
**V191I**	0.31	0.82	0.43	-0.45	0.59	-0.20	-1.11	-0.15	Possibly damaging
**A195T**	0.03	0.85	0.37	-0.96	-0.79	-0.96	4.06	0.35	Probably damaging
**M196V**	0.03	0.41	-0.37	-0.97	-1.11	-1.08	4.19	0.13	Benign
**A206T**	0.29	0.59	2.01	-1.05	-1.72	-0.99	-0.16	-0.38	Probably damaging
**M220L**	0.08	0.85	0.27	0.16	0.40	0.47	0.14	0.29	Benign
**R260Q**	0.25	0.15	-1.37	-0.49	-0.26	-0.28	0.23	-0.43	Benign
**I295V**	0.09	0.88	0.55	-1.04	-0.59	-0.97	0.50	-0.31	Possibly damaging
**P335R**	0.32	0.38	-3.49	0.15	1.18	0.40	1.85	0.02	Possibly damaging
**R363C**	0.54	0.88	2.53	-0.17	0.16	-0.10	0.29	0.54	Benign
**R363H**	0.54	0.88	2.00	-0.98	-0.41	-0.99	0.29	-0.02	Probably damaging
**T364M**	0.25	0.85	0.59	-0.03	0.30	-0.32	-0.62	-0.02	Probably damaging
**R367C**	0.56	0.32	3.65	-0.37	0.83	-0.30	0.28	0.82	Probably damaging
**H390T**	0.36	0.85	0.49	1.55	1.47	1.48	-0.34	0.93	Benign
**G425S**	0.12	0.56	1.13	-0.68	2.54	-0.28	-0.10	0.52	Benign
**A452T**	0.32	0.32	1.32	-1.28	-2.72	-1.18	0.41	-0.69	Possibly damaging
**G456S**	0.33	0.94	-0.43	-1.30	-2.55	-1.21	4.05	-0.29	Probably damaging
**R461C**	0.54	0.71	3.71	-0.39	0.16	-0.31	1.25	0.88	Probably damaging

Table S2. KNN classifications using different properties and K values. True positive (TP), true negative (TN) and accuracy are calculated for each K value.

Using rSASA, EC score, PD and ΔΔG				Using only rSASA, EC score, PD			
K	TP	TN	Accuracy	K	TP	TN	Accuracy
1	4	2	0.6	1	5	4	0.9
2	4	2	0.6	2	5	4	0.9
3	5	2	0.7	3	6	4	1
4	4	2	0.6	4	6	3	0.9
5	5	2	0.7	5	6	4	1
6	5	2	0.7	6	6	4	1
7	5	2	0.7	7	6	4	1
8	5	3	0.8	8	6	4	1
9	5	2	0.7	9	6	4	1
10	5	0	0.5	10	5	4	0.9

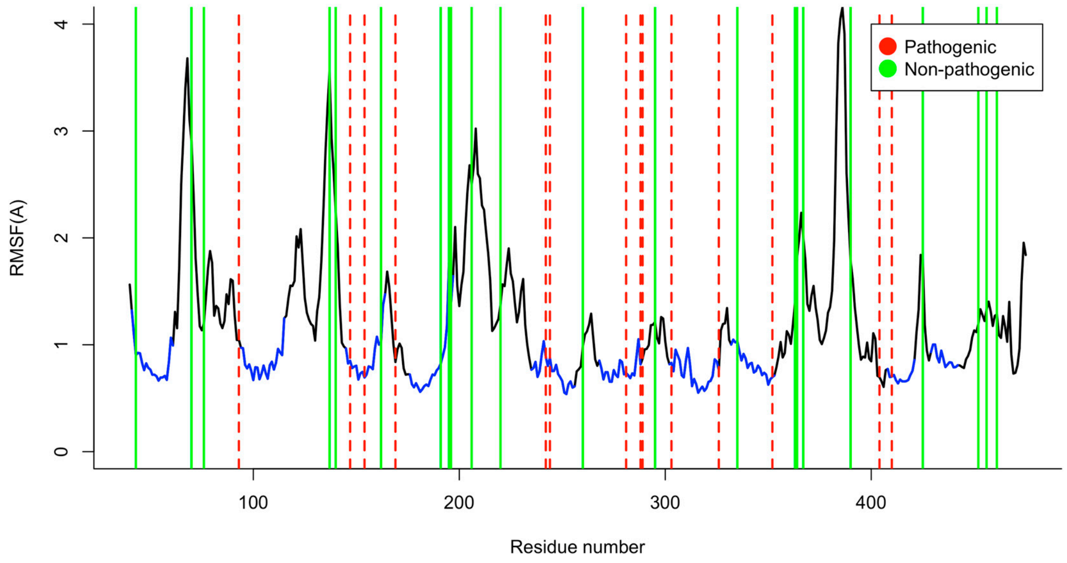

Figure 1. The pathogenic and non-pathogenic mutation occurring sites mapping on the average RMSF of the wild type DHCR7 protein. Pathogenic and non-pathogenic mutation sites are marked with red and green lines. The RMSF of transmembrane region are shown in blue.

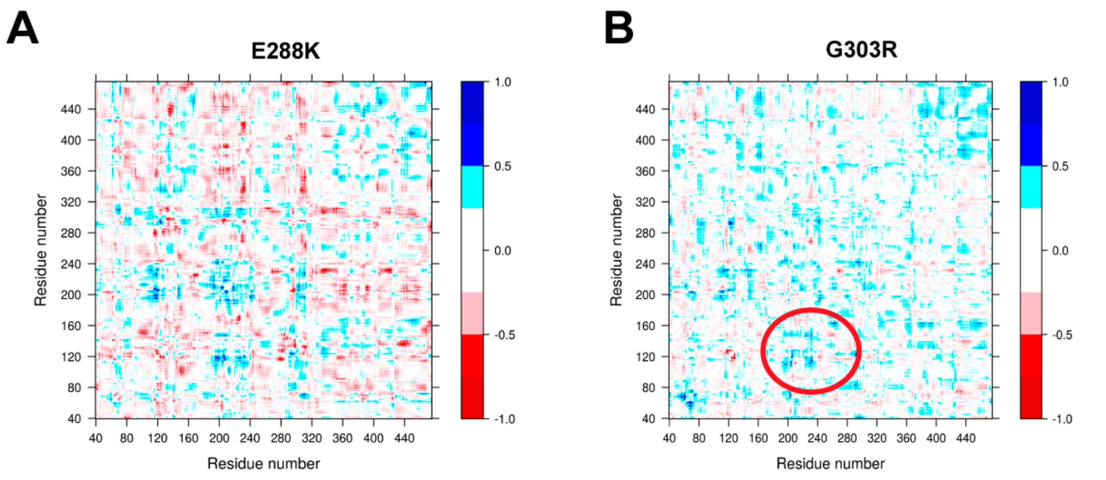

Figure S2. The changes in residue cross-correlation for mutations E288K and G303R.

```
DHCR7             MAAKSQPNIPKAKSLDGVTNDRTASQGQWGRAWEVDWFSLASVIFLLLFAPFIVYYFIMACDQYSCALTGPVVDIVTGHA
4QUV              MSEQE--------SRDNAAVDAVRQKYGFGFSWLV----------LMIALPPLVYYLWICVTYYQGELV-----FTSDAA
                  *: :.        * *..: * . .:  :* :* *          *::  * :***: :.   *.  *.     :.:. *

DHCR7             RLSDIWAKTPPITRKAAQLYTLWVTFQVLLYTSLPDFCHKFLPGYVGGIQEGAVTPAGVVNKYQINGLQAWLLTHLLWFA
4QUV              AWRRFWSHVAPPTWHAAGLYAAWFLGQAALQVWAP-----------GPTVQGMKLPDGSRLDYRMNGIFSFLFTLAVVFG
                      :*::..* * :** **: *.  *. * .  *           *   :*   * *   .*::**: ::*:*  : *.

DHCR7             NAHLLSWFSPTIIFDNWIPLLWCANILGYAVSTFAMVKGYFFPTSARDC-KFTGNFFYNYMMGIEFNPRIGKWFDFKLFF
4QUV              LV-TMGWLDATVLYDQLGPLLTVVNIFTFVFAGFL----YFWGLNGKQWERPTGRPFYDYFMGTALNPRIGS-LDLKLFC
                   .  :.*:..*:::*:  ***  .**: :..: *     **:  ..::  : **. **:*:**  :*****. :*:***

DHCR7             NGRPGIVAWTLINLSFAAKQRELHSHVTNAMVLVNVLQAIYVIDFFWNETWYLKTIDICHDHFGWYLGWGDCVWLPYLYT
4QUV              EARPGMIFWLLMNLSMAAKQYELHGTVTVPMLLVVGFQSFYLIDYFIHEEAVLTTWDIKHEKFGWMLCWGDLVWLPFTYT
                  :.***:: * *:***:**** ***. ** .*:**  :*::*:**:* :*   *.* ** *::*** * *** ****: **

DHCR7             LQGLYLVYHPVQLSTPHAVGVLLLGLVGYYIFRVANHQKDLFRRTDGRCLIWGRKPKVIECSYTSADGQRHHSKLLVSGF
4QUV              LQAQYLVHHTHDLPVWGIIAIVALNLAGYAIFRGANIQKHHFRRDP-NRIVWGKPAKYIKT--------KQGSLLLTSGW
                  **. ***:*. :*..   :.:: *.*.** *** ** **. ***   . ::**: .* *:         :: * **.**:

DHCR7             WGVARHFNYVGDLMGSLAYCLACGGGHLLPYFYIIYMAILLTHRCLRDEHRCASKYGRDWERYTAAVPYRLLPGI-
4QUV              WGIARHMNYFGDLMIALSWCLPAAFGSPIPYFHIVYFTILLLHREKRDDAMCLAKYGEDWLQYRKKVPWRIVPKIY
                  **:***:**.**** :*::**... *  :***:*:*::*** **  **:  * :***.** :*   **:*::* *
```

Figure S3. Sequence alignment between DHCR7 and template 4QUV.
